# Anticipatory and consummatory effects of (hedonic) chocolate intake are associated with increased circulating levels of the orexigenic peptide ghrelin and endocannabinoids in obese adults

**DOI:** 10.3402/fnr.v59.29678

**Published:** 2015-11-04

**Authors:** Antonello E. Rigamonti, Fabiana Piscitelli, Teresa Aveta, Fiorenza Agosti, Alessandra De Col, Silvia Bini, Silvano G. Cella, Vincenzo Di Marzo, Alessandro Sartorio

**Affiliations:** 1 Department of Clinical Sciences and Community Health, University of Milan, Milan, Italy; 2 Endocannabinoid Research Group, Institute of Biomolecular Chemistry, Consiglio Nazionale delle Ricerche, Pozzuoli, Italy; 3 Istituto Auxologico Italiano, IRCCS, Experimental Laboratory for Auxo-endocrinological Research, Milan and Verbania, Italy; 4 Istituto Auxologico Italiano, IRCCS, Division of Metabolic Diseases, Verbania, Italy

**Keywords:** palatable food, ghrelin, PYY, GLP-1, endocannabinoids, obesity, hunger, satiety

## Abstract

**Background:**

Hedonic hunger refers to consumption of food just for pleasure and not to maintain energy homeostasis. Recently, consumption of food for pleasure was reported to be associated with increased circulating levels of both the orexigenic peptide ghrelin and the endocannabinoid 2-arachidonoyl-glycerol (2-AG) in normal-weight subjects. To date, the effects of hedonic hunger, and in particular of chocolate craving, on these mediators in obese subjects are still unknown.

**Methods:**

To explore the role of some gastrointestinal orexigenic and anorexigenic peptides and endocannabinoids (and some related congeners) in chocolate consumption, we measured changes in circulating levels of ghrelin, glucagon-like peptide 1 (GLP-1), peptide YY (PYY), anandamide (AEA), 2-AG, palmitoylethanolamide (PEA), and oleoylethanolamide (OEA) in 10 satiated severely obese subjects after consumption of chocolate and, on a separate day, of a non-palatable isocaloric food with the same bromatologic composition. Evaluation of hunger and satiety was also performed by visual analogic scale.

**Results:**

The anticipatory phase and the consumption of food for pleasure were associated with increased circulating levels of ghrelin, AEA, 2-AG, and OEA. In contrast, the levels of GLP-1, PYY, and PEA did not differ before and after the exposure/ingestion of either chocolate or non-palatable foods. Hunger and satiety were higher and lower, respectively, in the hedonic session than in the non-palatable one.

**Conclusions:**

When motivation to eat is generated by exposure to, and consumption of, chocolate a peripheral activation of specific endogenous rewarding chemical signals, including ghrelin, AEA, and 2-AG, is observed in obese subjects. Although preliminary, these findings predict the effectiveness of ghrelin and endocannabinoid antagonists in the treatment of obesity.

Eating may be pleasurable and rewarding. In fact, food intake is motivated not only by the need to restore energy homeostasis; palatable, rewarding high-fat and/or sugar foods such as chocolate can motivate eating despite a state of satiety and positive energy balance. This phenomenon has been defined as ‘hedonic hunger’ in contraposition with ‘homeostatic hunger’, which is essentially triggered by energy deprivation (1).

Reportedly, obesity reflects an energy imbalance in which genetically susceptible individuals become increasingly vulnerable to an ‘obesogenic’ environment. Thus, both the palatability and availability of foods in the Western diet play a major role in the development of this (dramatically widespread) disease (2). An emerging hypothesis concerns the role of the brain's reward system that responds to the stimulus provided by rewarding and palatable ‘obesogenic’ foods and appears to override the homeostatic signals for body weight control (3). Indeed, mismatch between the hedonic value attributed to food and energy needs is characteristic of eating disorders, including (morbid) obesity. Therefore, understanding the physiological and pathophysiological mechanisms underlying the hedonic hunger may help to counteract obesity.

Animal data support the view that distinguishable although overlapping neural and peripheral pathways, involving several appetite-regulating substances, drive homeostatic- and hedonic-based eating (4, 5). Importantly, anticipatory effects to food are brain mediated, while changes in peripheral hormones are a related consequence.

Several gastrointestinal endocrine cells produce and secrete satiety hormones in response to food consumption and digestion. These hormones, including cholecystokinin (CCK), peptide YY (PYY), and glucagon-like peptide 1 (GLP-1), suppress homeostatic hunger and promote satiation and satiety mainly through hindbrain circuits, thus governing meal-by-meal eating behavior. Additionally, the hypothalamus integrates adiposity signals, specifically leptin, to regulate long-term energy balance and body weight. Distinct hypothalamic areas and various orexigenic and anorexigenic neurons have been identified to homeostatically regulate food intake. The hypothalamic circuits regulate food intake in part by modulating the sensitivity of the hindbrain to short-term satiety hormones (6).

In contrast, the hedonic and incentive properties of foods and food-related cues are processed by the cortico-limbic reward circuits. The mesolimbic dopamine system encodes subjective ‘liking’ and ‘wanting’ of palatable foods, which is subjected to modulation by the hindbrain and the hypothalamic homeostatic circuits and by satiety and adiposity hormones (6). A role for the orexigenic stomach-derived peptide ghrelin in mediating reward processes has also been demonstrated (7, 8). Particularly, the ghrelin receptor, GHS-R1a, is expressed not only in the hypothalamus but also in tegmental and mesolimbic areas involved in reward, such as the ventro tegmental area (VTA) and laterodorsal tegmental areas (LDTg); furthermore, intra-VTA or intra-LDTg administration of ghrelin increases accumbal dopamine release (7).

The endocannabinoids anandamide (AEA) and 2-arachidonoyl-glycerol (2-AG) are two lipid mediators that play a major role in the stimulation of food intake. They exert this function by activating cannabinoid type 1 (CB_1_) receptors, which are widely distributed in several brain areas, including those involved in the homeostatic and hedonic control of feeding (9, 10). Under normal physiological conditions in rodents, hypothalamic and limbic forebrain endocannabinoids transiently increase after food deprivation and decrease after food ingestion, possibly due to stimulatory or inhibitory effects by hormones whose circulating levels are modulated by food deprivation, such as ghrelin and leptin. These changes have been described to occur also in the human plasma (9).

Based on these considerations, one might hypothesize that endocannabinoid and gastrointestinal (orexigenic and anorexigenic) peptide responses to highly pleasurable food should differ from those to non-palatable food, in order to drive the motivation to eat even when there is no negative energy imbalance and, thus, to promote adipose tissue accumulation and obesity. Recently, Monteleone et al. (11, 12) showed that, in normal-weight healthy subjects, the consumption of food for pleasure was associated with increased circulating levels of both ghrelin and 2-AG, and that this response is disrupted in women with anorexia nervosa. These intriguing results should be confirmed and extended also to patients with obesity to better understand, in a pathophysiological context, the phenomenon of hedonic eating, which could powerfully influence food intake and, ultimately, body mass. In particular, chocolate consumption is a paradigm of hedonic eating that is often used in animal models of hyperphagia and obesity, but, as far as we know, its effect on endocannabinoid levels in humans has not been investigated. This is an important issue also in view of previous reports that have discussed whether or not the hedonic properties of chocolate can be ascribed in part to the presence in this food of very low amounts of endocannabinoids and related lipids (13, 14).

Thus, in order to explore the role of endocannabinoids and gastrointestinal peptides, specifically, ghrelin, PYY, and GLP-1, in hedonic eating in the obese state, we have measured here changes in the circulating levels of these mediators before and after the consumption of chocolate in satiated severely obese adults.

## Materials and methods

### Subjects

Ten male obese subjects, aged 19–44 yr (mean±SD=33.9±9.0 yr), having a mean BMI±SD 42.6±3.5 kg/m^2^, were enrolled into the study. The obese subjects were recruited from the Division of Metabolic Diseases at Istituto Auxologico Italiano, IRCCS, Verbania, Italy.

Each participant enrolled in the study was requested to fulfil the following conditions:

1. to positively respond to the following question: *Is chocolate one of your most favorite foods that you would eat also when satiated, just for pleasure?* (This is different from the protocol by Monteleone et al. (12), where the question was: *What is your most favourite food that you would eat also when satiated, just for pleasure?* – where chocolate was not always the answer.)
2. to give a palatability score ≥8 for chocolate, being the administered scale ranging from 0 (not-palatable) to 10 (maximally palatable).

Exclusion criteria included previous diagnosis of any disease affecting the endocrine system and metabolism (apart from obesity), chronic use of medications affecting metabolism and/or appetite, ≥5.0 kg weight change during the 3 months preceding study participation, allergies to or stated dislike of the components of the test meal (see below), and clinically diagnosed eating disorder or a score of ≥20 on the eating attitudes test (15). No subject was a marijuana smoker, an alcohol consumer, or heavy cigarette smoker, which are conditions known to affect circulating levels of endocannabinoids.

The experimental protocol was approved by the local ethical committee and all subjects gave their written consent after being fully informed of the nature and procedures of the study. Therefore, each subject was aware that, in the first session of the experimental protocol, he would have eaten chocolate.

### Study design

The experiment used a within-subject repeated-measure design in which each volunteer served as his own control, similar to that used by Monteleone et al. (12). All subjects were tested two times with an interval in-between the tests of at least 7 days. A single-blind, Latin-square crossover design could not be applied because of the experimental needs of evoking the anticipatory effect of palatable food and of administering a non-palatable food with the same nutrients (isobromatologic) and calorie (isoenergetic) amounts of the consumed palatable food (see below). The anticipation to food intake consisted in two periods: 1) when obese subjects were informed about the (palatable/non-palatable) food they would have eaten (T0–T60) and 2) when obese subjects could have sensorial experiences related to the (palatable/non-palatable) food (T60–T70).

On the first test session, participants arrived at our Clinical Investigation Unit at 08:30 h after a 12-h fast. At 09:00 h, they were asked to rate their hunger and satiety on visual analog scales (VAS) that used a 10-cm line with labels at the extremities indicating the most negative and the most positive ratings; immediately afterward, an intravenous catheter was inserted into an antecubital vein to collect a first blood sample (time (*T*)=0); the catheter was connected to a saline solution, which was slowly infused to keep it patent through the entire experimental session. Then, the subjects received a breakfast of 300 kcal, with 77% carbohydrates, 10% proteins, and 13% fats. Immediately after breakfast (consumed within 10 min), they rated again their hunger and satiety by means of VAS. Further blood samples were drawn (T10 and T30 at 10 min and 30 min, respectively). After 1 h from the start of the study, the subjects were told that they would receive chocolate. Immediately afterwards, each participant was exposed to the palatable food for 10 min. During this time, he could smell and see the food but could not eat it. At the end of the exposure, each participant was asked to rate his hunger, satiety by means of VAS. Blood samples were drawn at 60 min and 70 min (i.e. T60 and T70). Then, the subject was free to eat the palatable food (see below for details) within 10 min. Additional blood samples were drawn immediately after the exposure to the palatable food (T80) and at 100 min (T100), 130 min (T130), 160 min (T160), and 190 min (T190); at the same time points, they rated again their hunger and satiety by means of VAS.

At the end of the session, the amount of food eaten by each participant was calculated by weighing the residual food and subtracting it from the initial amount of food provided, and then the calories eaten were calculated.

On the second test session, carried out at least 7 days later, participants underwent the same experimental procedures of the first experimental session except for the fact that they were exposed to non-palatable food and had to eat an amount of it with the same nutrient composition and an equal quantity of calories (i.e. isobromatologic and isoenergetic) as the palatable food they ate in the previous session within 10 min.

During the food exposure (specifically, from T60 to T70), a total of 20 pictures of chocolate-based foods and of landscapes and nature were shown in the session with chocolate and non-palatable foods, respectively.

### Palatable and non-palatable foods

The palatable food was a milk-chocolate tablet (200 g for a total of 1,000 kcal with 61.4% carbohydrates, 7.9% proteins, and 30.7% fat), served in a dish from which the subject was free to eat until he became satiated (for a maximum corresponding to the whole chocolate tablet).

The non-palatable food, which was identified by all participants as non-desirable just for pleasure (specifically, with a palatability score <2) consisted of bread and butter, which were combined *ad hoc* to provide the same nutrients (isobromatologic) and calorie (isoenergetic) amounts of the consumed chocolate. Calorie and nutrient contents of palatable and non-palatable foods were calculated by using the information reported on the labels of each packaged foods (chocolate tablet and butter). To calculate calorie and nutrient content of bread, we obtained the recipe from the baker who made it.

To maintain a stable daily caloric intake of the in-hospital obese patients, the amount of foods administered at lunch and dinner of the experimental days was proportionally reduced to account for the calories of the test meals (i.e. chocolate or non-palatable food).

### Evaluation of body composition

Anthropometric characteristics were evaluated during the screening period. BMI was calculated from measured height and weight. Fat-free mass (FFM) and fat mass (FM) were evaluated by the bioelectrical impedance analysis (Human-IM Scan, DS-Medigroup, Milan, Italy).

### Blood sampling and biochemical measurements

Blood was collected in tubes with or without anticoagulant (EDTA). Plasma or serum was separated by centrifugation and stored at −20° C.

Total plasma ghrelin level, including both octanoylated and des-octanoylated ghrelin, was measured by a commercially available RIA for ghrelin (Millipore, Saint Charles, MO). The sensitivity of the method was 93 pg/ml; intra- and interassay coefficients of variation (CVs) were 10.0 and 14.7%, respectively.

Total plasma PYY level, including both PYY1-36 and PYY3-36, was measured by a commercially available RIA for PYY (Millipore). The sensitivity of the method was 10 pg/ml; intra- and interassay CVs were 2.9 and 7.1%, respectively.

Total plasma GLP-1 level, including GLP-17-36 amide, GLP-17-37, GLP-19-36 amide, GLP-19-37, GLP-11-36 amide, and GLP-11-37, was measured, after an extraction procedure, by RIA (Millipore). A DPP-4 inhibitor was added to tubes to prevent the breakdown of GLP-1. The sensitivity of the method was 3 pmol/l; intra- and interassay CVs were 2.9 and 7.1%, respectively.

Serum insulin concentration was determined by chemiluminescent immunometric assay using a commercial kit (Immulite 2000, DPC, Los Angeles, CA). The sensitivity of the method was 2 µIU/ml; intra- and interassay CVs were 22–38 and 14–23%, respectively.

Serum glucose level was measured by the glucose oxidase enzymatic method (Roche Diagnostics, Monza, Italy).

Plasma levels of AEA, 2-AG, oleoylethanolamide (OEA), and palmitoylethanolamide (PEA) were determined by isotopic dilution-liquid chromatography–mass spectrometry as described previously (15, 16).

### Statistical analysis

The Sigma Stat 3.5 statistical software package was used for data analysis. GraphPad Prisma 5.0 software was used for plotting data. The Shapiro–Wilk test showed that all parameters were normally distributed.

Results are reported as mean±SD (standard deviation). The responses in glucose, insulin, ghrelin, PYY, GLP1, AEA, 2-AG, PEA, OEA, and VAS scores for hunger and satiety were evaluated as absolute values and also as area under the curve (AUC) of postprandial measurements using the trapezoid rule for each experimental session of eating (breakfast+chocolate and breakfast+non-palatable with AUC_0–190_ for ghrelin, GLP-1, PYY, VAS scores for hunger and satiety, glucose and insulin, and AUC_60–190_ for AEA, 2-AG, PEA, and OEA).

All parameters (ghrelin, PYY, GLP-1, VAS scores for hunger and satiety, glucose and insulin) were compared within each experimental session of eating (breakfast+chocolate and breakfast+non-palatable food) over sampling times (intra-group analysis) and between the two experimental sessions of eating for any sampling time (inter-group analysis) by using a two-way ANOVA with repeated measures (with the two factors: time and session and the interaction time×session), followed by the *post-hoc* Tukey's test, which was used to compare responses after breakfast (i.e. T10, T30, and T60 vs. 0 min) and the responses after chocolate or non-palatable food (i.e. T80, T100, T130, T160, and T190 vs. 70 min) for both experimental sessions of eating (i.e. breakfast+chocolate and breakfast+non-palatable food). The same statistical test was applied for analyzing the responses in endocannabinoids (AEA, 2-AG, PEA, and OEA) only after the second part of the experimental session (i.e. T70, T100, T130, and T190 vs. 60 min for both experimental sessions of eating). Student's *t*-test was used to compare AUCs of each peptide or endocannabinoids in both experimental sessions. Pearson's product-moment correlation test (for all data) was employed to analyze possible correlations among the variables. A level of significance of *p*<0.05 was used for all data analyses.

## Results

### Body composition and other clinical information

The percent FM was 41.2±5.1%, and the percent FFM was 58.9±5.1% (Table 1).

**Table 1:** Demographic and clinical characteristics of the obese subjects enrolled in the study.

	Obese subjects
Number (no.)	10
Age (yr)	33.4±9.0
BMI (kg/m^2^)	42.9±3.5
FFM (kg)	78.9±12.6
FFM (%)	58.9±5.1
FM (kg)	54.8±7.8
FM (%)	41.2±5.1

BMI=body mass index; FM=free fat mass; FM=fat mass.

No statistically significant differences in body weight were found in all obese subjects between the first (breakfast+chocolate) and second (breakfast+non-palatable food) sessions of eating (data not shown).

### Calorie ingestion

No statistically significant difference emerged in the mean values of calories and nutrients of palatable and non-palatable foods (data not shown), confirming the precision of dietetic calculations (see above). Obese subjects ate 152.4±48.5 g of chocolate (range: 74–200 g), which corresponds to 787.1±230.6 kcal (range: 396–1,000 kcal).

### Circulating levels of gastrointestinal peptides: ghrelin, PYY, and GLP-1

#### Ghrelin

**Fig. 1:**
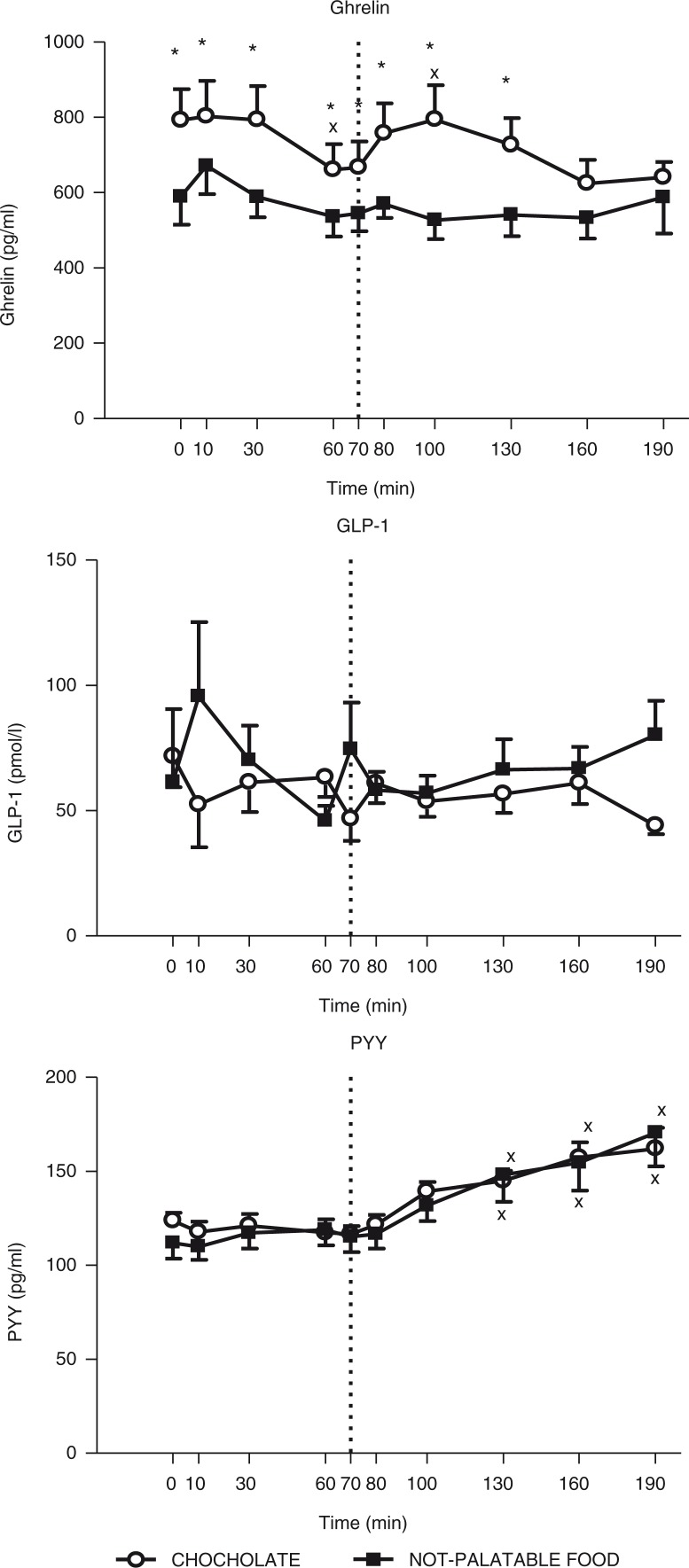
Changes of circulating levels of ghrelin (top panel), GLP-1 (middle panel) and PYY (bottom panel) in obese subjects after breakfast (at the left of the dotted vertical line, i.e. T0–T70) and chocolate or non-palatable meal (at the right of the dotted vertical line, i.e. T70–T190) during the hedonic and non-palatable sessions of eating, respectively. Breakfast was consumed from T0 to T10, while chocolate or non-palatable meal was consumed from T70 to T80 after a sensorial exposure of the foods and view of pictures of chocolate-based foods (in the hedonic session) or landscapes and nature (in the non-palatable session) from T60 to T70. See the text for further details. Values are expressed as mean±SD. *p<0.05 vs. the corresponding time point of the non-palatable session; ×*p*<0.05 vs. the corresponding T0 or T70 value.

The time×session repeated-measures ANOVA yielded significant main effects for time (*F*(9)=4.36, *p*<0.01) and session (*F*(1)=19.41, *p*<0.05), without any significant interaction for time×session (*F*(9)=1.65), indicating that circulating levels of ghrelin changed significantly over sampling times and between the experimental sessions of eating (i.e. administration of breakfast+chocolate vs. breakfast+non-palatable food). Indeed, the *post-hoc* Tukey's test indicated that 1) there was a statistically significant decrease in circulating levels of ghrelin at 60 min (vs. 0 min after the breakfast, *p*<0.01) in obese patients administered with the breakfast+chocolate session, without any difference in those administered with the breakfast+non-palatable session (intra-group analysis) and 2) the obese subjects tested with the hedonic session, when compared with the non-palatable session, had significantly higher plasma concentrations of ghrelin at all times (*p*<0.01), except for 160 min and 190 min (inter-group analysis) (Fig. 1).

#### PYY

The time×session repeated-measures ANOVA yielded a significant main effect for time (*F*(9)=2.04, *p*<0.01), without any significant effect for session (*F*(1)=0.05) and interaction for time×session (*F*(9)=0.53), indicating that circulating levels of PYY changed significantly over the sampling times, but not between the two experimental sessions of eating. Indeed, the *post-hoc* Tukey's test indicated that administration of both experimental sessions of eating (i.e. breakfast+chocolate or breakfast+non-palatable food) evoked an identical statistically significant increase in circulating levels of PYY at 130 min, 160 min, and 190 min (vs. 70 min after hedonic or non-palatable food, *p*<0.01) (intra-group analysis) (Fig. 1).

#### GLP-1

The time×session repeated-measures ANOVA yielded no significant main effects for time (*F*(9)=0.40) and session (*F*(1)=0.64) and no significant interaction for time×session (*F*(9)=1.67), indicating that circulating levels of GLP-1 did not change significantly over sampling times and between the two experimental sessions of eating (Fig. 1).

### Circulating levels of endocannabinoids and related mediators: AEA, 2-AG, PEA, and OEA

#### AEA

**Fig. 2:**
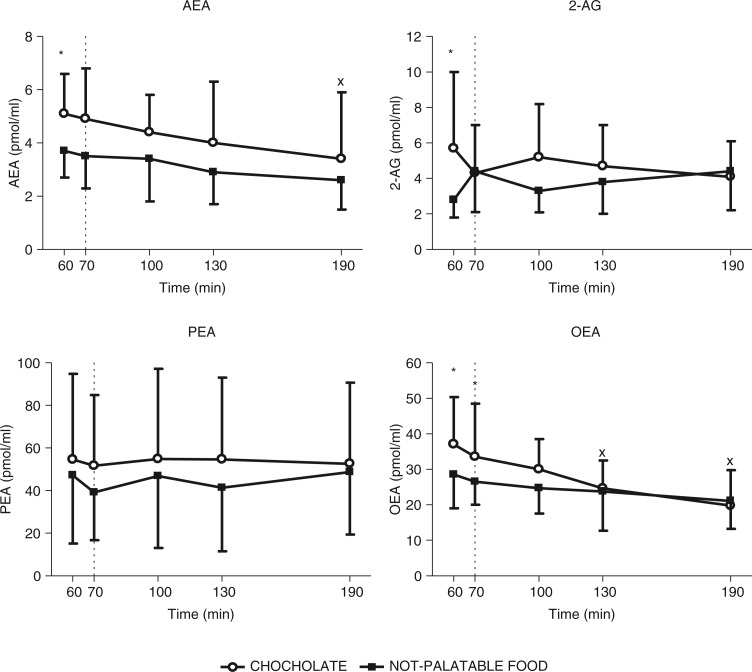
Changes of circulating levels of anandamide (AEA, top left panel), 2-arachidonoyl-glycerol (2-AG, top right panel), palmitoylethanolamide (PEA, bottom left panel), and oleoylethanolamide (OEA, bottom right panel) in satiated obese subjects before (i.e. T60–T70) and after (i.e. T60–T190) chocolate or non-palatable meal during the hedonic and non-palatable sessions of eating, respectively. Chocolate or non-palatable meal was consumed from T70 to T80 after a sensorial exposure of the foods and view of pictures of chocolate-based foods (in the hedonic session) or landscapes and nature (in the non-palatable session) from T60 to T70. See the text for further details. Values are expressed as mean±SD. *p<0.05 vs. the corresponding time point of the non-palatable session; ×*p*<0.05 vs. the corresponding T60 value.

The time×session repeated-measures ANOVA yielded significant main effects for time (*F*(4)=4.19, *p*<0.01) and session (*F*(1)=7.42, *p*<0.05), without any significant interaction for time×session (*F*(4)=0.21), indicating that circulating levels of AEA changed significantly over sampling times and between the experimental sessions of eating (i.e. administration of breakfast+chocolate vs. breakfast+non-palatable food). Indeed, the *post-hoc* Tukey's test indicated that 1) there was a statistically significant decrease in circulating levels of AEA at 190 min (vs. 60 min, *p*<0.05) in obese patients administered with the breakfast+chocolate session, without any difference in those administered with the breakfast+non-palatable-food session (intra-group analysis) and 2) the obese subjects tested with the hedonic session, when compared with the non-palatable session, had significantly higher plasma concentrations of AEA at 60 min (*p*<0.05) (inter-group analysis) (Fig. 2).

#### 2-AG

The time×session repeated-measures ANOVA yielded a significant main effect for session (*F*(1)=1.40, *p*<0.05), without any significant main effect for time (*F*(4)=0.11) and interaction for time×session (*F*(4)=3.00), indicating that circulating levels of 2-AG did not change significantly over sampling times, but did between the experimental sessions of eating. Indeed, the *post-hoc* Tukey's test indicated that 1) there was no statistically significant difference in circulating levels of 2-AG in obese patients administered with the breakfast+chocolate or breakfast+non-palatable-food session (intra-group analysis) and 2) the obese subjects tested with the hedonic session, when compared with the non-palatable session, had significantly higher plasma concentrations of 2-AG at 60 min (*p*<0.05) (inter-group analysis) (Fig. 2).

#### PEA

The time×session repeated-measures ANOVA yielded no significant main effects for time (*F*(4)=0.11) and session (*F*(1)=1.33) and no significant interaction for time×session (*F*(4)=0.17), indicating that circulating levels of this non-endocannabinoid AEA homolog did not change significantly over sampling times and between the experimental sessions of eating (Fig. 2).

#### OEA

The time×session repeated-measures ANOVA yielded significant main effects for time (*F*(4)=12.97, *p*<0.01) and session (*F*(1)=2.24, *p*<0.05), without any significant interaction for time×session (*F*(4)=1.18), indicating that circulating levels of this non-endocannabinoid AEA homolog changed significantly over sampling times and between the experimental sessions of eating. Indeed, the *post-hoc* Tukey's test indicated that 1) there was a statistically significant decrease in circulating levels of OEA at 130 min and 190 min (vs. 60 min, *p*<0.05) in obese patients administered with the breakfast+chocolate session, without any difference in those administered with the breakfast+non-palatable-food session (intra-group analysis) and 2) the obese subjects tested with the hedonic session, when compared with the non-palatable session, had significantly higher plasma concentrations of OEA at 60 min and 70 min (*p*<0.05) (inter-group analysis) (Fig. 2).

### VAS scores: hunger and satiety

#### Hunger

**Fig. 3:**
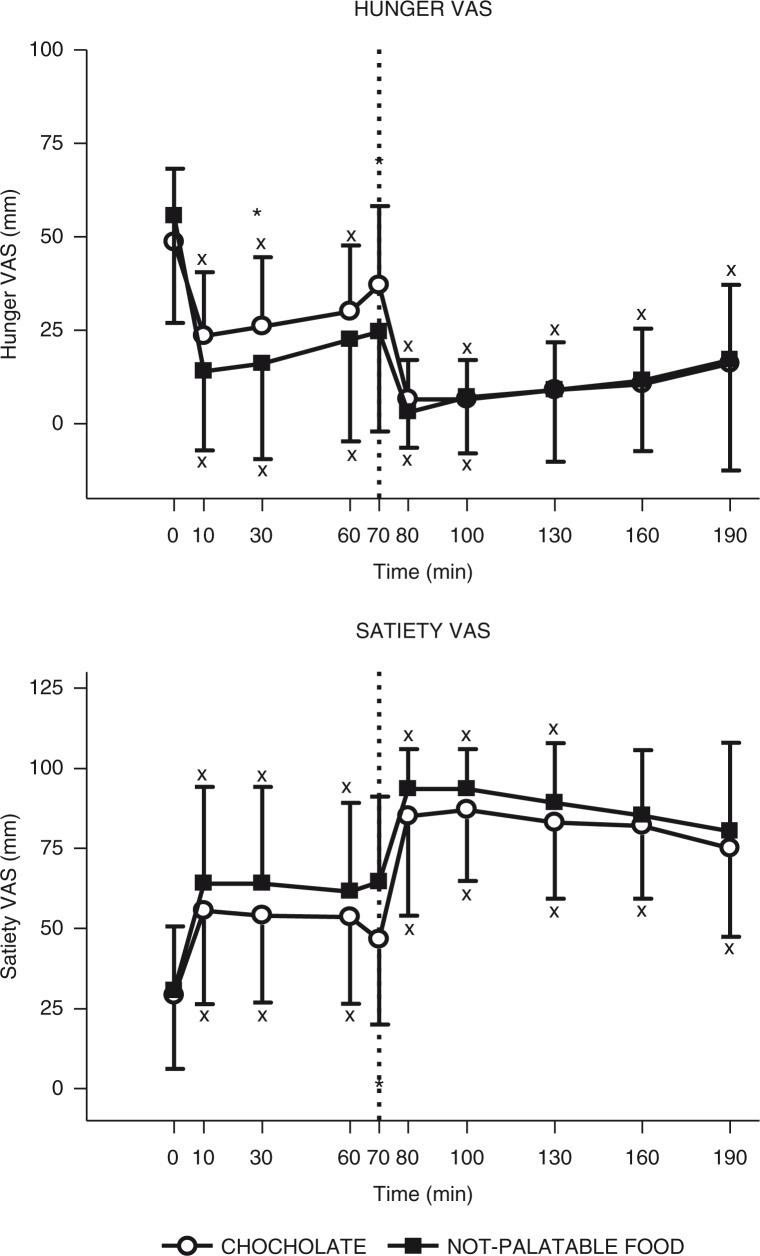
Changes of VAS ratings of hunger (top panel) and satiety (bottom panel) in obese subjects after breakfast (at the left of the dotted vertical line, i.e. T0–T70) and chocolate or non-palatable meal (at the right of the dotted vertical line, i.e. T70–T190) during the hedonic and non-palatable sessions of eating, respectively. Breakfast was consumed from T0 to T10, while chocolate or non-palatable meal was consumed from T70 to T80 after a sensorial exposure of the foods and view of pictures of chocolate-based foods (in the hedonic session) or landscapes and nature (in the non-palatable session) from T60 to T70. See the text for further details. Values are expressed as mean±SD. *p<0.05 vs. the corresponding time point of the non-palatable session; ×*p*<0.05 vs. the corresponding T0 or T70 value.

The time×session repeated-measures ANOVA yielded a significant main effect for time (*F*(9)=15.92, *p*<0.01) and interaction for time×session (*F*(9)=2.98, *p*<0.01), without any significant main effect for session (*F*(1)=0.58), indicating that hunger VAS score changed significantly over sampling times and between the experimental sessions of eating (i.e. administration of breakfast+chocolate vs. breakfast+non-palatable food). Indeed, the *post-hoc* Tukey's test indicated that 1) there was a statistically significant decrease in hunger VAS scores at 10 min, 30 min, and 60 min (vs. 0 min after the breakfast, *p*<0.01) for both experimental sessions and at 80 min and 100 min (vs. 70 min after the chocolate or non-palatable foods in obese patients administered with the breakfast+chocolate and the breakfast+non-palatable-food sessions, respectively, and also at 130 min, 160 min, and 190 min (vs. 70 min after chocolate) only in the hedonic session (intra-group analysis) and 2) the obese subjects tested with the hedonic session, when compared with the non-palatable session, had significantly higher values in hunger VAS score at 30 min and 70 min (*p*<0.01) (inter-group analysis) (Fig. 3).

#### Satiety

The time×session repeated-measures ANOVA yielded significant main effects for time (*F*(9)=25.80, *p*<0.01) and for session (*F*(1)=1.42, *p*<0.01), without any interaction for time×session (*F*(9)=0.58), indicating that satiety VAS score changed significantly over sampling times and between the experimental sessions of eating. Indeed, the *post-hoc* Tukey's test indicated that 1) there was a statistically significant increase in satiety VAS scores at 10 min, 30 min, and 60 min (vs. 0 min after the breakfast, *p*<0.01) for the breakfast+chocolate and breakfast+non-palatable-food sessions and at 100 min and 130 min (vs. 70 min after the chocolate or non-palatable food) for both sessions and also at 160 min and 190 min (vs. 70 min after the chocolate) only for the breakfast+chocolate session (intra-group analysis); 2) the obese subjects tested with the hedonic session, when compared with the non-palatable session, had significantly lower values in satiety VAS score at 70 min (*p*<0.01) (inter-group analysis) (Fig. 3).

### Metabolic parameters: glucose and insulin

#### Glucose

**Fig. 4:**
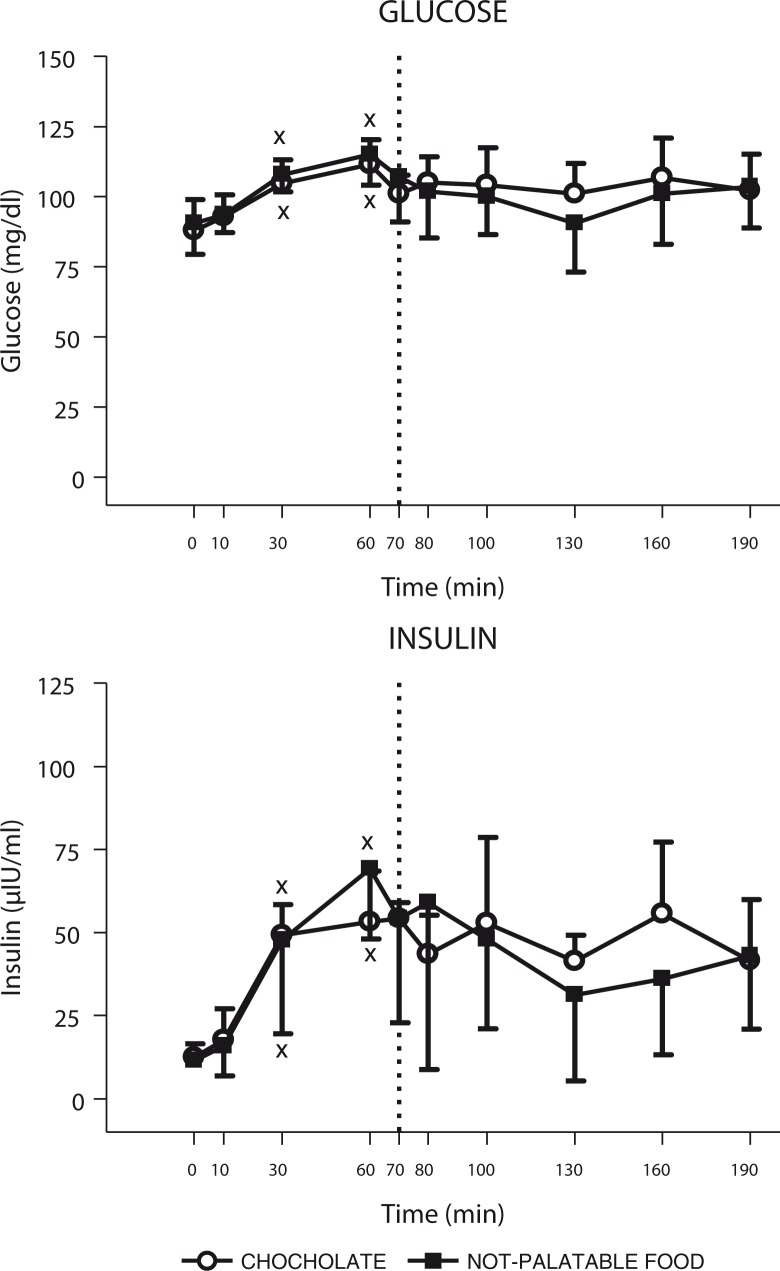
Changes of circulating levels of glucose (top panel) and insulin (bottom panel) in obese subjects after breakfast (at the left of the dotted vertical line, i.e. T0–T70) and chocolate or non-palatable meal (at the right of the dotted vertical line, i.e. T70–T190) during the hedonic and non-palatable sessions of eating, respectively. Breakfast was consumed from T0 to T10, while chocolate or non-palatable meal was consumed from T70 to T80 after a sensorial exposure of the foods and view of pictures of chocolate-based foods (in the hedonic session) or landscapes and nature (in the non-palatable session) from T60 to T70. See the text for further details. Values are expressed as mean±SD. *p<0.05 vs. the corresponding time point of the non-palatable session; ×*p*<0.05 vs. the corresponding T0 or T70 value.

The time×session repeated-measures ANOVA yielded a significant main effect for time (*F*(9)=10.93, *p*<0.01), without any significant effect for session (*F*(1)=3.68) and interaction for time×session (*F*(9)=1.52), indicating that glucose concentrations changed significantly over the sampling times, but not between the two experimental sessions of eating (i.e. administration of breakfast+chocolate vs. breakfast+non-palatable food). Indeed, the *post-hoc* Tukey's test indicated that administration of both experimental sessions of eating (i.e. breakfast+chocolate or breakfast+non-palatable foods) evoked an identical statistically significant increase in glucose concentrations at 30 min and 60 min (vs. 0 min after breakfast, *p*<0.01) (intra-group analysis) (Fig. 4).

#### Insulin

The time×session repeated-measures ANOVA yielded a significant main effect for time (*F*(9)=7.42, *p*<0.01), without any significant effect for session (*F*(1)=0.13) and interaction for time×session (*F*(9)=1.11), indicating that insulin concentrations changed significantly over the sampling times, but not between the two experimental sessions of eating. Indeed, the *post-hoc* Tukey's test indicated that administration of both experimental sessions of eating (i.e. breakfast+chocolate or breakfast+non-palatable foods) evoked an identical statistically significant increase in insulin concentrations at 30 min and 60 min (vs. 0 min after breakfast, *p*<0.01) (intra-group analysis) (Fig. 4).

### AUCs of circulating levels of peptides, endocannabinoids, and related mediators, of metabolic parameters and of VAS scores for hunger and satiety

The AUC of circulating levels of ghrelin was significantly higher in obese subjects administered with the breakfast+chocolate session than with the breakfast+non-palatable-food session. No statistically significant differences were found for AUCs of circulating levels of GLP-1 and PYY. The AUC of circulating levels of AEA was significantly higher in obese subjects administered with the breakfast+chocolate session than with the breakfast+non-palatable-food session. No statistically significant differences were found for AUCs of circulating levels of 2-AG, PEA, and OEA. No statistically significant differences were found for VAS scores of hunger and satiety and concentrations of glucose and insulin (Table 2).

**Table 2:** Areas under the curve (AUC) of circulating levels of peptides, endocannabinoids, and metabolic parameters and of VAS scores for hunger and satiety.

	Palatable	Not-palatable	*p*
Ghrelin (pg/ml×min)	110488.2±9905.7	87651.1±8611.8	*p*<0.05
GLP-1 (pmol/l×min)	8619.9±727.5	11929.3±1294.6	–
PYY (pg/ml×min)	23449.6±1208.8	23142.5±2188.2	–
AEA (pmol/ml×min)	535.0±225.7	374.0±73.1	*p*<0.05
2-AG (pmol/ml×min)	599.1±211.8	468.2±184.4	–
PEA (pmol/ml×min)	6995.1±4806.0	5409.6±3001.2	–
OEA (pmol/ml×min)	3458.7±1055.5	2905.8±862.4	–
Hunger VAS (mm×min)	2002.5±2118.0	1875.0±3064.3	–
Satiety VAS (mm×min)	12612.5±3643.7	13616.0±2966.3	–
Glucose (mg/dl×min)	16600.0±1408.7	15672.5±2061.4	–
Insulin (µIU/ml×min)	8007.6±3732.5	7676.1±4072.7	–

AUC_0-190_ for ghrelin. PYY, GLP-1, VAS scores for hunger and satiety, glucose, and insulin. AUC_60-190_ for AEA, 2-AG, PEA, and OEA.

### Correlations

Among the most relevant correlations, the AUC of circulating levels of ghrelin was correlated with that of OEA (*r*=0.46, *p*<0.05); that of GLP-1 with that of 2-AG (*r*=−0.45, *p*<0.05); and that of AEA with that of PEA (*r*=0.74, *p*<0.01). Interestingly, the AUC of hunger VAS score was correlated with that of AEA (*r*=0.68, *p*<0.05) and that of PEA (*r*=0.58, *p*<0.01). Values of BMI were correlated with AUCs of circulating levels of AEA (*r*=−0.60, *p*<0.01), 2-AG (*r*=0.49, *p*<0.05), and PEA (*r*=−0.55, *p*<0.05). Values of FM (kg) were correlated with AUCs of circulating levels of GLP-1 (*r*=−0.51, *p*<0.05), AEA (*r*=−0.41, *p*<0.05), and PEA (*r*=−0.54, *p*<0.01), while those of FM (%) with the AUC of PEA (*r*=−0.39, *p*<0.05).

## Discussion

Monteleone et al. (12), while investigating the regulation of circulating levels of ghrelin and endocannabinoids after hedonic eating in satiated normal-weight subjects, have recently shown that the consumption of food for pleasure was associated with increased circulating levels of both ghrelin and 2-AG. In contrast, plasma levels of both AEA and of the two AEA metabolically related lipids and agonists of peroxisome proliferator-activated receptor α (PPAR-α), PEA and OEA progressively decreased after the ingestion of both palatable and (isoenergetic and isobromatologic) non-pleasurable food.

In the present study, higher circulating levels of ghrelin were persistently found in obese subjects during the entire hedonic session (breakfast+palatable-food session), in which, 1 h after a breakfast, a chocolate tablet was initially served for a complete sensorial experience and then freely consumed; in contrast, a stable lower profile in circulating levels of ghrelin was found during the breakfast+non-palatable-food session, in which the chocolate was substituted for an isoenergetic and isobromatologic non-palatable meal (bread and butter). In addition, during the (first part of the) hedonic session, a significant inhibition of ghrelin secretion occurred after breakfast, which was unable to reduce circulating levels of the same orexigenic peptide when obese subjects were being included in the non-palatable session. Finally, the experience with the palatable food (i.e. the second part of the session with the direct exposure to chocolate, view of pictures of chocolate-based foods and consumption of the chocolate tablet) significantly further increased ghrelin secretion, whereas there were no changes in circulating levels of this peptide during the last part of the non-palatable session, in which the non-palatable meal was served in conjunction with the view of pictures of landscapes and nature and, entirely, consumed.

In our experimental protocol, satiation was obtained by eating a breakfast of 300 kcal. Although this could seem an energy amount not enough to completely suppress hunger in obese subjects fasted from 12 h, eating such an amount of calories at breakfast is in line with Italian feeding habits and, therefore, better represents the natural morning feeding condition in our participants.

The unexpected breakfast-suppressible hyperghrelinemia in our obese subjects, tested in the hedonic session of eating, was already present at T0 before starting the experiment. This is in contrast with the low circulating levels and the lack of any post-meal suppression of ghrelin in obesity as demonstrated by the obese subjects tested with the non-palatable session of the present study and many other works (17, 18).

In our obese subjects, the ghrelin increase observed in plasma before starting the experiment and also at T100 after administration of the palatable meal likely reflects the stimulation of ghrelin secretion occurring in the cephalic phase (or, alternatively, the anticipatory phase) of ingestion of highly palatable food, when any (lean or obese) individual thinks, sees, and/or smells the food but does not eat it yet (19). The anticipatory effect of highly palatable food is, obviously, a crucial aspect of our experimental protocol: specifically, the participants in the present study knew that they would have eaten the highly pleasurable food in the first experimental session, because of the need to balance the energy amounts and bromatologic composition of the non-palatable food to that of the palatable one.

However, at the beginning of the hedonic session of eating and after the exposure to the palatable food, such ghrelin increases (at T0 and T100) were (relatively) more pronounced in the obese subjects enrolled in the present study when compared with those observed in the normal-weight subjects included in the study by Monteleone et al. (12), in which a similar protocol was adopted. Based on these findings, one might argue that obese subjects, who, similarly to normal-weight subjects (12), were aware that they would have eaten a palatable food in that day before starting the experiment and, actually, had an intense sensorial exposure to the palatable food (T60–T70), are more sensitive to any food-related cues than normal-weight subjects, with the consequence of a (relatively) more pronounced stimulation of ghrelin secretion. This secretory pattern of ghrelin was not present in obese subjects during the non-palatable session, in which the profile of circulating levels of ghrelin remained depressed over the entire duration of the protocol. As previously mentioned, this latter secretory pattern of ghrelin is that more known and commonly described in most works dealing with the effects of food intake on circulating levels of this peptide (17, 18), without differentiating administration of palatable and non-palatable foods, which, as demonstrated by the present study, is crucial to induce hyperghrelinemia.

A functional magnetic resonance imaging (fMRI) study (20) showed that intravenous ghrelin administration in normal-weight subjects increases the neural response to food pictures in brain areas implicated in reward processing and appetitive behaviour, such as the amygdala, ventral striatum, anterior insula, and orbitofrontal cortex (OFC). Moreover, experimental data demonstrated that injection of ghrelin into the third ventricle of mice significantly increases locomotor activity as well as extracellular dopamine levels in the nucleus accumbens (7), a neurochemical system involved in reward and motivated behavior as well as in mediating the incentive salience of food (21). There is some evidence that activation of these neuroanatomic areas may be influenced by abnormal ghrelin levels associated with genetic risk for obesity or obese weight status (22).

Following exogenous administration of PYY_3–36_ and GLP-1_7–36__amide_, both independently and in combination, in fasted normal-weight individuals, reductions were observed in neural activity in the striatum, insula, and OFC in response to palatable food images (23), suggesting downstream inhibitory effects of these hormones on the same regions stimulated by ghrelin. Therefore, obese individuals, having lower circulating PYY and insensitivity to GLP-1 (24, 25), would have, at least theoretically, an increased activity in the striatum, insula, and OFC during exposure to food.

As expected, in the present study, circulating levels of PYY and GLP-1 were low and only a slow progressive increase in PYY secretion was found after the second part of the two sessions of eating, without any difference between consumptions of palatable and non-palatable meals.

So, if ghrelin-induced activation of the mesolimbic dopamine reward system increases the incentive value of food and facilitates food-seeking behavior (7), on the basis of our data in obese subjects, it can be tentatively speculated that an increased secretion of ghrelin, which, similarly to normal-weight subjects (12), precedes and ensues the consumption of the palatable food, potently activates central reward pathways, which, differently from normal-weight subjects (23), are not inhibited (or are only weakly) by PYY and GLP-1. Therefore, in obese subjects, who usually have low (homeostatic and also protective) ghrelin levels (17, 18), when exposed to palatable food, eating is promoted not only by the anticipatory increase in ghrelin secretion, but also by the insufficient anorexigenic responses of PYY and GLP-1. Despite no need for calorie ingestion, obese subjects eat just for the rewarding properties of the highly pleasurable food, with no or insufficient anorexigenic brake.

This view is confirmed by other data of the present study, that is, 1) the efficacy of breakfast to reduce and increase, respectively, the hunger and the satiety in both sessions of eating (i.e. breakfast+palatable-meal and breakfast+non-palatable-meal), indicating the induction of a similar state of satiation with no need for calorie ingestion, irrespective from the palatability of the next meal and 2), importantly, the ability of the palatable food to reduce less and increase less, respectively, the hunger and the satiety (at 70 min) in the hedonic session of eating (i.e. breakfast+palatable-meal).

The present study suggests the importance of palatable-food-related cues, mainly sensorial ones, to stimulate food intake in obese subjects. This appears to be mediated by ghrelin in lean and obese subjects, but actually amplified in obesity, with potential long-term detrimental effects in an environment offering unlimited amounts of palatable food. Administration of ghrelin antagonists, which has been considered by some authors as an inappropriate therapeutic intervention because of low ghrelin levels present in obese subjects (26, 27), might represent, in light of these preliminary results, a valid solution to block the rewarding effects of the hyperghrelinemia preceding any exposure to palatable food and ingestion of excessive amounts of hypercaloric foods in conditions of positive energy balance. The same result might be obtained with psychotherapy, aimed to desensitize the ‘food-addicted’ subject from palatable-food-related cues. Further studies are mandatory to investigate the therapeutic potential of these interventions in obesity.

The findings of the present study also suggest an involvement of the endocannabinoids AEA and 2-AG, and of the non-endocannabinoid AEA congener OEA (see also below), in the modulation of hedonic eating. Specifically, in our obese subjects, circulating levels of AEA, 2-AG, and OEA were significantly higher at T60, before the exposure to chocolate, being OEA the only endocannabinoid that was also higher at T70, after exposure to chocolate tablet and view of pictures of chocolate-based foods, but just before the consumption of this palatable food. Furthermore, the AUC of circulating levels of AEA was significantly higher in obese subjects administered with chocolate than non-palatable food. Finally, there was a significant decrease of circulating levels of AEA and OEA only at the end of the breakfast+chocolate session (i.e. at T190), without any differences for the other endocannabinoids 2-AG and PEA in the same session and for all endocannabinoids in breakfast+non+palatable-food session.

These data suggest an activation of endogenous production of AEA, 2-AG, and OEA before exposure to chocolate, an effect which is similar to the anticipatory increase in ghrelin levels in hedonic eating, as discussed above. Combination of increases in ghrelin and endocannabinoids (at least, the well-known rewarding CB_1_ agonists AEA and 2-AG) would promote the ‘wanting to eat’ and also the ‘liking eating’ in a condition of no energy deprivation (28). Accordingly, in the present study, hunger VAS score was positively correlated with circulating levels of AEA (and PEA). A limit of this argumentation is the missing measurement of endocannabinoids before T60.

Data in laboratory animals suggest that exposure to foods with high salience and incentive properties stimulate an endocannabinoid tone to induce dopamine release in the limbic area (9). This might, in turn, lead to both increased motivation to consume palatable foods (also when there is no need for calorie ingestion) (i.e. wanting to eat) and heighten rewarding effects after the consumption of such foods (i.e. liking eating). Therefore, the increased plasma levels of AEA and 2-AG that we found in obese subjects exposed to the palatable food might be the result of spill-over from the brain areas in the reward system. Alternatively, circulating levels of these endocannabinoids might reflect spill-over from peripheral tissues, such as the small intestine and/or adipose tissue, which, like the brain, respond to food deprivation and refeeding with changes in local endocannabinoid levels (29–31). The possibility of a brain-driven control of endocannabinoid production in peripheral tissues may not be ruled out; thus, the anticipatory effects of a highly palatable food, which are centrally mediated, might influence spill-over of endocannabinoids at a peripheral level from the small intestine or adipose tissue. Further studies are mandatory to determine whether the increased circulating levels of endocannabinoids reflect changes in peripheral tissues or in brain areas directly involved in reward. This might be important to characterize new pharmacologic targets for obesity.

In the work by Monteleone et al. (12), conducted in normal-weight subjects who underwent a protocol similar to ours, a positive correlation was found between ghrelin levels and those of 2-AG, both measured as AUC, suggesting an interaction between ghrelin and the endocannabinoid system. In accordance with some studies in laboratory animals (32–34), it has been proposed that the role of peripheral ghrelin in the rewarding effects of highly pleasurable food is mediated by an activation of the endogenous production of endocannabinoids, particularly 2-AG, or vice versa.

As demonstrated by the present study, in obese subjects there was no correlation between ghrelin and 2-AG or the other well-known rewarding CB_1_ agonist AEA, but between ghrelin and OEA. To date, little evidence for a role for PPAR-α and the two PPAR-α ligands, OEA and PEA, in the rewarding effect of food has been reported, apart from a single study, which pointed to counter-rewarding actions for these compounds (35). Therefore, it is difficult to interpret this correlation as well as the finding of higher circulating levels of OEA (but not PEA) in obese subjects exposed to chocolate, when compared to those in breakfast+non-palatable-food session. This is in contrast with the study by Monteleone et al. (12), in which, in normal-weight subjects, the levels of OEA and PEA were similar in both groups with palatable and non-palatable foods, suggesting a causative role of the obese state for the unique secretory pattern of OEA in our obese subjects. Further studies in animals are mandatory to understand the physiological role and the pathophysiological implications of the PPAR-α agonists, including the endogenous PEA and OEA, in food intake and, generally, reward.

Monteleone et al. (12) found significant decreases in AEA, 2-AG, PEA, and OEA after meal consumption, irrespectively from the palatability of the food. Although insulin has been reported to reduce circulating levels of AEA and 2-AG in a way inversely related to anthropometric and metabolic predictors of insulin resistance and dyslipidemia, without any specific information for PEA and OEA (36), in the present study, the finding of unchanged circulating levels of 2-AG and PEA after administration of palatable food and of all endocannabinoids after non-palatable food is possibly due to the inability of insulin to inhibit the biosynthesis, or up-regulation of the degradation, of these compounds in our insulin-resistant obese subjects (36). This explanation may be challenged by the decline of circulating levels of AEA after ingestion of palatable food, which, however, was very slow and possibly due to potential circadian changes in the levels of this compound, previously described in the rat brain (37). On the contrary, the exception of OEA, whose circulating levels decreased after ingestion of chocolate (but not non-palatable food), seems to confirm the unique secretory pattern of this presumably not-rewarding endocannabinoid in obese subjects exposed to palatable food.

Before closing, some important aspects should be mentioned.

Due to the experimental design adopted in the present study (i.e. fixed order, with subjects knowing ahead what was coming *per* session), we cannot exclude systematic order effects. We chose this design based on the argument of the experimental needs of evoking the anticipatory effect of palatable food and of administering a non-palatable isocaloric and with the same macronutrient composition as the consumed palatable food (chocolate). This conditioning paradigm cannot be accomplished by adopting any alternative design with randomized order (e.g. administration of fixed portions, so that palatable and non-palatable foods could have been matched on energy and macronutrient content beforehand).

We have compared above our results in obese subjects with those obtained in normal-weight subjects by Monteleone et al. (12). However, although these authors used a similar protocol, they employed a different type of hedonic food. In fact, the subjects recruited in the present study were asked to eat chocolate as one of their favorite foods, whereas in the previous study, normal-weight subjects were asked to select their most favorite food, which was not necessarily chocolate. The possibility that the exclusive use of chocolate in this study may have been a source of differences in the observed results should not be underestimated for at least two reasons: 1) chocolate is reported to contain centrally acting compounds, which, if reaching the brain after consumption, might explain the rewarding properties of this food together with its unique sensory properties, and might also modify (i.e. repress or stimulate) both central or peripheral endocannabinoid biosynthesis; 2) chocolate, especially milk chocolate, has been described to contain endocannabinoids, albeit in trace levels, as well as compounds such as OEA (13, 14). This latter fact, however, is unlikely to have affected the measured plasma levels of AEA, 2-AG, and OEA, which were found here to be higher only before (T70) and/or immediately after (T70) the consumption of chocolate *vs*. non-palatable food. Furthermore, very low amounts of dietary endocannabinoids and acylethanolamides are likely to be immediately degraded in the gastrointestinal tract before being adsorbed. However, possible modulatory actions of cocoa psychoactive components, such as caffeine, theobromine, and phenylethylamine, on the biosynthesis of these lipid mediators may have affected their measured amounts at later time points.

Finally, we enrolled only a limited number of obese male subjects. Women are known to have more preference to chocolate than men (e.g. 91% of women crave on chocolate vs. 59% of men (38). Thus, we expect that obese female subjects will have an exaggerate ghrelin response to palatable food, specifically chocolate. Further studies are mandatory to investigate this (potential) gender-related difference and to confirm the preliminary results obtained in the present study.

In conclusion, possibly similar to normal-weight subjects (12), when motivation to eat is generated by the availability of highly palatable food and not just by food deprivation, a peripheral activation of specific endogenous rewarding chemical signals, including ghrelin, AEA, and 2-AG, is observed in obese subjects. However, when compared to that in normal-weight subjects, this response seems to be amplified, especially for what concerns ghrelin, with no anorexigenic post-meal responses in GLP-1 and PYY. After the early withdrawal of rimonabant, a CB_1_ inverse agonist originally used to decrease body weight in obesity, from the pharmaceutical market because of safety concerns, the present preliminary study may predict the effectiveness of ghrelin receptor and CB_1_ neutral antagonists in the treatment of hyperphagia and bingeing on highly palatable foods in obesity.
